# Exploring the Synthesis of Lactic Acid from Sugarcane Molasses Collected in Côte d’Ivoire Using *Limosilactobacillus fermentum* ATCC 9338 in a Batch Fermentation Process

**DOI:** 10.3390/bioengineering12080817

**Published:** 2025-07-29

**Authors:** Asengo Gerardin Mabia, Harinaivo Anderson Andrianisa, Chiara Danielli, Leygnima Yaya Ouattara, N’da Einstein Kouadio, Esaïe Kouadio Appiah Kouassi, Lucia Gardossi, Kouassi Benjamin Yao

**Affiliations:** 1 Joint Research and Innovation Unit in Agronomic Sciences and Transformation Processes (UMRI SAPT), Laboratory of Industrial Processes of Synthesis, Environment and New Energies (LAPISEN), Institut National Polytechnique Félix Houphouët-Boigny, Yamoussoukro BP 1093, Côte d’Ivoire; leygnima.ouattara18@inphb.ci (L.Y.O.); nda.kouadio20@inphb.ci (N.E.K.); appiah.kouassi@inphb.ci (E.K.A.K.); benjamin.yao@inphb.ci (K.B.Y.); 2 Department of Chemistry and Agricultural Industries, Institut Facultaire des Sciences Agronomiques de Yangambi (IFA-Yangambi), Kisangani BP 1232, Democratic Republic of the Congo; 3 Laboratoire Eau, Hydro-Systèmes et Agriculture (LEHSA), International Institute for Water and Environmental Engineering (2iE), Ouagadougou 01 BP 594, Burkina Faso; anderson.andrianisa@2ie-edu.org; 4 Department of Chemical and Pharmaceutical Sciences, University of Trieste, Via L. Giogieri 1, 34127 Trieste, Italy; chiara.danielli@units.it (C.D.); gardossi@units.it (L.G.)

**Keywords:** lactic acid, molasses valorisation, *Limosilactobacillus fermentum*, cost reduction, fermentation, optimisation, design of experiments

## Abstract

Lactic acid (LA) is a high-value chemical with growing demand for the production of polymers and plastics and in the food and pharmaceutical industries. However, production costs remain a significant constraint when using conventional food-grade substrates. This study investigates Ivorian sugarcane molasses, an abundant agro-industrial by-product, as a low-cost carbon source for LA production via batch fermentation with *Limosilactobacillus fermentum* ATCC 9338. Molasses was pretreated by acid hydrolysis to improve fermentability, increasing glucose and fructose concentrations. Comparative fermentations using raw and pretreated molasses showed a 75% increase in LA production (32.4 ± 0.03 g/L) after pretreatment. Optimisation using Box–Behnken design revealed that the initial sugar concentration, inoculation rate, and stirring speed significantly influenced lactic acid production. Under optimal conditions, a maximum LA concentration of 52.4 ± 0.49 g/L was achieved with a yield of 0.95 g/g and productivity of 0.73 g/L·h. Kinetic analysis confirmed efficient sugar utilisation under the optimised conditions, and polarimetry revealed a near-racemic lactic acid. A simplified cost analysis showed that molasses could reduce carbon source costs by over 70% compared to refined sugars, supporting its economic viability. This work demonstrates the potential of pretreated molasses under robust fermentation conditions as a sustainable and cost-effective substrate for LA production in resource-limited contexts. The approach aligns with circular bioeconomy principles and presents a replicable model for decentralised bioproduction in a developing country like Côte d’Ivoire.

## 1. Introduction

Lactic acid (2-hydroxypropanoic acid) is a versatile organic acid with many industrial applications, including in the food, pharmaceutical, and biodegradable plastic industries [1]. The increasing demand for lactic acid, particularly in the production of poly(lactic acid) (PLA), which is a promising bio-based material that is biocompatible, transparent, and thermoplastic, making it a strong candidate for replacing conventional plastics, has led to a significant need for cost-effective and sustainable production methods [2,3]. Notably, the LA structure features a chiral carbon atom, and the (L)-(+)-enantiomer with an (S)-configuration is widely used in the production of renewable materials that have replaced a significant proportion of conventional plastics in food packaging. The biodegradability of PLA is negatively affected by the crystallinity of the polymer, which is highest in stereohomogeneous polymers composed of a sequence of PLA molecules with the same absolute configuration [4].

The global PLA market is expected to grow rapidly due to increased environmental awareness and regulatory pressure [2]. However, the high cost of producing lactic acid, particularly when derived from refined sugars and food-based substrates, remains a significant barrier to market expansion [3]. Conventional lactic acid fermentation often relies on starch or glucose derived from corn or sugarcane, raising concerns about food security, raw material cost volatility, and ethical concerns [1]. Consequently, agro-industrial residues have attracted growing attention as low-cost, non-food feedstocks for bioproduction processes [5,6].

Among these, sugarcane molasses, a viscous byproduct of sugar refining, is particularly promising due to its high fermentable sugar content, year-round availability, and low market value [7,8,9]. Several studies have explored its use as a substrate for the microbial production of value-added products such as lactic acid [1,10,11,12], bioethanol [13], succinic acid [14], biosurfactants [15], and enzymes [7]. Its richness in sugars (typically 40–60% *w*/*v*) and micronutrients (e.g., potassium, calcium, and nitrogenous compounds) makes it an ideal candidate for microbial fermentation without requiring extensive supplementation [8,16]. Moreover, molasses can support the growth of a wide variety of microorganisms, including *Lactobacillus* spp., *Saccharomyces cerevisiae*, and *Bacillus* spp. [10,11,17]. Valorising molasses aligns with circular economy principles by converting waste streams into high-value chemicals while mitigating environmental disposal issues.

Recent advances highlight the capacity of lactic acid bacteria (LAB) to metabolise a variety of agro-industrial residues for the production of lactic acid and other value-added biochemicals. Select strains of *Bifidobacterium* and *Lactobacillus* have demonstrated efficient carbohydrate utilisation and biopolymer synthesis when cultivated on lignocellulosic or sugar-rich agricultural waste streams, supporting their role in low-cost, substrate-flexible fermentation systems [18,19]. Balasubramanian et al. [19] further emphasised the role of renewable substrates in microbial lactic acid production for bioplastic synthesis, while Hussein et al. [18] demonstrated the capacity of local *Bifidobacterium* strains to produce exopolysaccharides from low-cost media, reinforcing the broader biotechnological potential of LAB and related microorganisms in agro-waste valorisation.

In Côte d’Ivoire, the sugar industry produces approximately 76,000 tonnes of molasses annually as a by-product of 200,000 tonnes of raw sugar production [8]. Despite this abundance, molasses remains underutilised, often discarded or sold for low-value purposes such as animal feed. Given its local abundance and inherent nutrient content, molasses could serve as a carbon and micronutrient source, reducing reliance on expensive fermentation additives such as yeast extract and peptone [20]. Recent local research efforts have explored the valorisation of molasses and other local agro-wastes. Studies have examined the chemical characterisation of Ivorian molasses, its use in ethanol production, and its combination with cashew apple juice to improve lactic acid yields [8,21]. Parallel research has also investigated alternative agricultural wastes such as cocoa pod husks for lactic acid production [22], indicating growing national interest in bioresource conversion. However, these studies are mostly preliminary, often lacking process optimisation, enantiomeric characterisation, or economic assessment.

This fragmented research landscape and continued disposal of molasses and agro-waste hampers waste management and compromises alignment with global sustainability goals. Without integrated bioprocessing strategies, valuable resources are lost and pollution increases. Hybrid approaches that combine chemical pretreatment with microbial conversion have shown promise for valorising complex substrates, as demonstrated in a study coupling catalytic and microbial degradation for waste transformation [23]. Similarly, developing a scalable, environmentally friendly fermentation process using sugarcane molasses in Côte d’Ivoire is a scientific and socio-environmental imperative.

The success of such a strategy depends on the ability of robust lactic acid bacteria (LAB) strains to tolerate variable substrate quality while maintaining high yields. Lactic acid bacteria, especially *Lactobacillus* strains, are widely used in bioprocessing for their ability to convert sugars into lactic acid under mild conditions [10,24,25,26]. *Limosilactobacillus fermentum* ATCC 9338, a heterofermentative strain, is known for its adaptability to diverse sugar profiles and moderate fermentation conditions [22,27,28]. This makes it suitable for molasses-based fermentation systems in regions with limited processing infrastructure.

This study investigates the feasibility of using Ivorian sugarcane molasses as a cost-effective carbon source for lactic acid production. This by-product, originating from a semi-industrial sugar sector characterised by localised agro-ecological practices and partially mechanised processing, exhibits a distinctive biochemical profile, with a particularly high sucrose content and relatively low concentrations of fermentation inhibitors. Unlike many molasses types from Asia, Australia, or South America, which often contain higher levels of inhibitors due to more intensive refining processes [29,30,31], these less intensive practices likely confer a composition that is highly desirable for lactic acid production.

Therefore, the objective of this study was to develop and optimise a fermentation process for lactic acid production using Ivorian sugarcane molasses as the primary substrate. The fermentation performance of *L. fermentum* ATCC 9338 was evaluated on both raw and pretreated molasses, with process conditions optimised via Box–Behnken design under response surface methodology. The specific rotational power of the lactic acid was assessed due to its importance in industrial applications, and comparative cost analysis was conducted to assess the economic feasibility of this process relative to conventional fermentation substrates. By targeting both process performance and local resource valorisation, this study contributes to sustainable biomanufacturing in West Africa.

## 2. Materials and Methods

### 2.1. Raw Material

**Figure 1:**
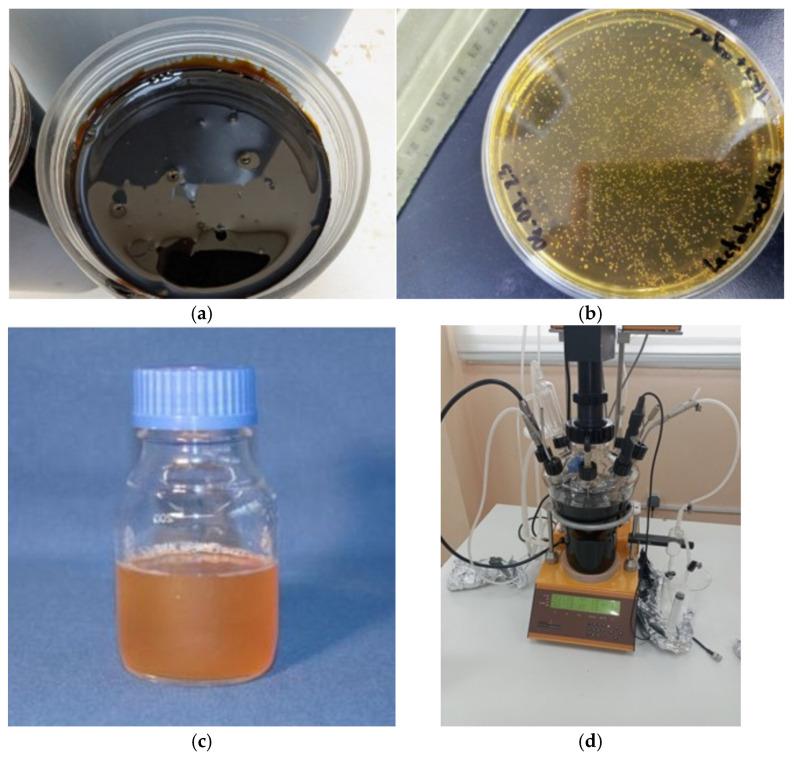
Key materials and equipment used in the fermentation process: (**a**) Raw sugarcane molasses; (**b**) *Limosilactobacillus fermentum* ATCC 9338 strain; (**c**) Prepared inoculum; (**d**) LAMBDA MINIFOR biofermentor.

The sugarcane molasses (SCM) used in this study was obtained from the Société Sucrière Africaine (SUCAF) sugar factory in Ferkessédougou, northern Côte d’Ivoire (9°32′ N, 6°29′ W). The molasses had a viscosity of 4798 cP and a density of 1.38 g/mL. Analytical grade sulphuric acid (H_2_SO_4_) and sodium hydroxide (NaOH) used for pretreatment and pH adjustment were supplied by Fisher Scientific (Waltham, MA, USA). The materials used for the fermentation experiments are shown in Figure 1, including raw sugarcane molasses, the *Limosilactobacillus fermentum* ATCC 9338 strain, the prepared inoculum, and the LAMBDA MINIFOR biofermentor.

### 2.2. Pretreatment of Sugarcane Molasses

To improve fermentability, SCM was subjected to a dilution and acidolysis pretreatment protocol with modification [32]. The molasses was diluted with distilled water in a ratio of 1:5 (*w*/*v*) and heated at 80 °C for 30 min in the presence of 1 N H_2_SO_4_. The pH was adjusted to 8.5 throughout the process with 4 M NaOH to prevent sugar degradation. After heating, the solution was cooled and neutralised to pH 5.5 for optimal fermentation conditions. This pretreatment aimed to hydrolyse sucrose into fermentable monosaccharides (glucose and fructose) and reduce the viscosity and inhibitory mineral content.

### 2.3. Determination of the SCM Composition

The composition of both raw and pretreated molasses was analysed to determine the concentrations of key carbohydrates (sucrose, fructose, and glucose) and other components. Carbohydrate concentrations were measured using spectrophotometric analysis following the phenol–sulfuric acid method described in [33], with absorbance measured at 490 nm using a Jenway 75862 spectrophotometer (Cole-Parmer Ltd., Stone, UK). Calibration curves were prepared using standard solutions of each sugar. Mineral concentrations (Ca, Mg, Fe, Cu, Mn, Na, K, Zn) were quantified by inductively coupled plasma atomic emission spectroscopy (ICP-AES) 20 type VARIAN, following the AOAC 984.27 method as improved by Poitevin et al. [34], with minor adaptations for the sugarcane molasses matrix. Samples were digested in nitric acid using microwave-assisted digestion prior to analysis. The crude protein content was calculated from the total nitrogen, determined by the Kjeldahl digestion method following ISO 1871:2009 [35], using a conversion factor of 6.25. These compositional data supported the selection of suitable fermentation conditions and helped to evaluate the effect of pretreatment on molasses quality.

### 2.4. Microorganism and Inoculum Preparation

*Limosilactobacillus fermentum* ATCC 9338, a heterofermentative LAB, was used as the production strain. It was purchased from Industrial Analytical (PTY, Johannesburg, South Africa) and maintained at −80 °C in MRS broth supplemented with 20% (*v*/*v*) glycerol to ensure long-term viability.

For activation, the strain was cultured in MRS broth at 37 °C with shaking at 200 rpm for 72 h according to the procedure described in [22]. Optical density (OD_600_) was monitored using a Jenway 75862 spectrophotometer.

### 2.5. Lactic Acid Production

Batch fermentations were initially carried out using raw and pretreated molasses to assess the substrate suitability. The diluted molasses was adjusted to an initial sugar concentration of 40 g/L for each experiment. The fermentations were carried out in a 3 L biofermentor (LAMBDA MINIFOR, LAMBDA Laboratory Instruments, Baar, Switzerland) with a 2 L working volume, operated at 37 °C, 200 rpm and an inoculation rate of 10% (*v*/*v*) using a cell density of approximately 2.38 × 10^6^ CFU/mL. The pH was maintained at 5.5 by automatic titration with 4 M NaOH.

Only pretreated molasses was used for optimisation. A Box–Behnken design (BBD) under response surface methodology (RSM) was used to investigate the effects of three independent variables: initial sugar concentration (g/L), inoculation rate (%), and stirring speed (rpm). The factor choice was based on preliminary experiments. The three independent factors used were investigated at three different levels, as shown in Table 1.

The experimental matrix consisted of 15 randomised runs, including 3 centres. Design Expert software (version 13, Stat-Ease Inc., Minneapolis, MN, USA) was used for statistical design and data analysis. This design allowed for the efficient exploration of the parameter space and the identification of optimal conditions for maximising lactic acid production. Fermentation experiments were conducted in a 3 L laboratory fermenter LAMBDA MINIFOR model, with a working volume of 2 L, and samples were taken every 8 h. Optical density, sugar concentration, and lactic acid content were monitored. After fermentation, cells were removed by centrifugation (6000 rpm, 15 min, 4 °C, HERMLE Z 326 K, HERMLE Labortechnik GmbH, Wehingen, Germany) and the supernatant was used for analysis.

### 2.6. Kinetic Parameters of Lactic Acid Production Process

The kinetic parameters of the lactic acid production process were calculated to evaluate fermentation performance. Volumetric lactic acid productivity (Q_p_, g/L/h) was calculated as the ratio of lactic acid concentration at the end of fermentation (P_t_, g/L) to the total fermentation time (t, h), as shown in Equation (1). The yield of lactic acid based on sugar consumed (Y_P/S_, g/g) was determined using Equation (2), where S_0_ and S_f_ (g/L) represent the initial and final sugar concentrations, respectively.

(1) $$Q_{P}=\frac{P_{t}}{t}$$

(2) $$Y_{P/S}=\frac{P_{f}}{S_{0}-S_{f}}$$

### 2.7. Cost Analysis

A simplified cost analysis was conducted to evaluate the economic feasibility of lactic acid production using pretreated sugarcane molasses as a cost-effective carbon source in Côte d’Ivoire. Data on the sugar content (% *w*/*w*), local price (USD/ton), and effective sugar cost (USD/kg) for each substrate were compiled from peer-reviewed scientific articles, local agro-industry reports, and publicly available market databases such as FAO statistics. For molasses, direct pricing was verified with the local industry. Where primary data were unavailable, estimates were derived based on regional biomass collection costs, pretreatment efficiencies, and reported hydrolysis yields. The effective sugar cost was calculated using Equation (3), ensuring a normalised comparison across substrates with different sugar contents. Sugar content values correspond to fermentable (monomeric) sugars after any necessary pretreatment, consistent with the compositional analysis reported in Section 3.1.

(3) $$\mathrm{Effective}\;\mathrm{sugar}\;\mathrm{cost}\;(\frac{\mathrm{USD}}{\mathrm{kg}})=\frac{\mathrm{Local}\;\mathrm{price}\;(\frac{\mathrm{USD}}{\mathrm{ton}})}{\mathrm{Sugar}\;\mathrm{content}\;\left(\%\right)\times 10}$$

The calculation did not include pretreatment costs (energy, chemicals), which are qualitatively discussed in the economic analysis. To ensure the robustness of the estimates, a range of values was reported based on the compositional variability found in previous studies. This approach provides a realistic and comparable basis for evaluating the economic feasibility of molasses and alternative substrates for lactic acid production under West African industrial conditions.

### 2.8. Analytical Methods

Batch lactic acid quantification was performed using a spectrophotometric method adapted from [36] based on the reaction between lactate ions and iron (III) chloride, which forms a coloured complex with an absorbance peak at 390 nm. Calibration curves were prepared using pure D/L-lactic acid (90%). For each condition, samples were centrifuged at 6000 rpm for 15 min, and the supernatant was analysed. A 50 μL aliquot of the supernatant was mixed with 2 mL of 0.2% iron (III) chloride, and the absorbance was measured within 15 min.

The specific rotational power was measured with a Jasco P-2000 polarimeter (JASCO Corporation, Tokyo, Japan), using a Jasco J/39.35 quartz cell (JASCO Corporation, Tokyo, Japan), with an optical path length of 100 mm. Optical rotation was measured in water at a concentration of 2.5 g/100 mL at 20 °C, as reported in the literature and confirmed by Sigma-Aldrich technical data ([α]_D_ = +2.6°) [37,38].

### 2.9. Statistical Analysis

All analyses were performed in triplicate, and the data were expressed as the mean ± standard deviation (SD). Analysis of variance (ANOVA) was performed using Design Expert version 13 to evaluate the significance of model terms, with *p*-values < 0.05 considered statistically significant. Model adequacy was assessed through R^2^, adjusted R^2^, predicted R^2^, and diagnostic residual plots. Fermentation kinetics plots were produced using OriginPro 2024 (OriginLab Corp., Northampton, MA, USA).

## 3. Results

### 3.1. Chemical Characterisation of Raw and Pretreated Ivorian Sugarcane Molasses

The compositional analysis of raw and pretreated molasses is shown in Table 2. The pretreatment significantly altered the chemical profile of the molasses, improving the availability of fermentable sugars. In particular, the sucrose content decreased sharply from 56.8 ± 2.5 g/L to 7.4 ± 0.7 g/L, while glucose and fructose concentrations increased to 31.5 ± 1.5 g/L and 33.8 ± 1.2 g/L, respectively. This confirms the effectiveness of the hydrolysis process in breaking down disaccharides into simpler sugars that are readily assimilated by *Limosilactobacillus fermentum* ATCC 9338 [28,32].

In parallel, reductions in total dissolved solids, ash content, and mineral concentrations (including calcium, magnesium, iron, copper, manganese, and zinc) were observed. These reductions are beneficial for microbial growth and metabolic efficiency, as a high mineral content, particularly heavy metals, can inhibit bacterial fermentation [39]. The reduction in pH from 5.7 to 5.1 also contributes to a more suitable acidic environment for lactic acid bacteria. Thus, the pretreatment not only increased the fermentable sugar content but also reduced inhibitory compounds, making the substrate more conducive to lactic acid fermentation. The chemical composition of the Ivorian molasses in this study showed slightly lower sugar and mineral values than those reported by [8] for the same site, probably due to seasonal variations in sugarcane quality and differences in storage or sampling time [16,30]. However, these values are still higher than those reported by [9] in other regions.

### 3.2. Lactic Acid Production from Raw and Pretreated Molasses

**Figure 2:**
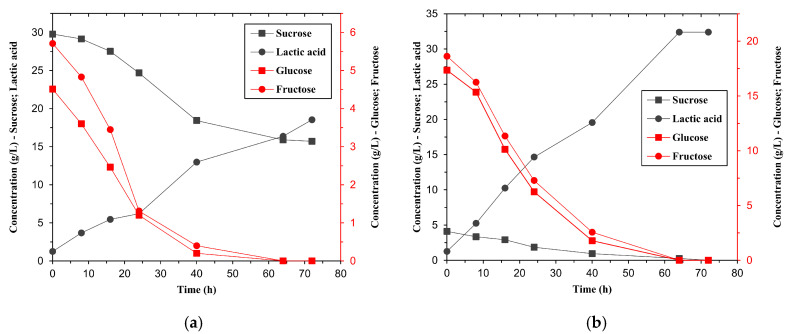
Profiles of sugar consumption during batch fermentation of (**a**) raw molasses and (**b**) pretreated molasses with *Limosilactobacillus fermentum* ATCC 9338 for the production of lactic acid.

Fermentation trials with raw and pretreated molasses under identical conditions showed a significant improvement in lactic acid production with pretreated substrates (Table 3). With raw molasses, lactic acid production reached 18.54 ± 0.05 g/L, whereas with pretreated molasses, it reached 32.42 ± 0.03 g/L, an increase of 75%. This improvement is attributed to the increased availability of monosaccharides (glucose and fructose) after sucrose hydrolysis, as supported by the sugar consumption profiles in Figure 2.

The sugar consumption profile shows a clear difference in utilisation: in raw molasses (Figure 2a), a significant amount of residual sucrose remained unutilised, whereas in pretreated molasses (Figure 2b), the sugars were completely consumed within 40 h. This demonstrates that although the strain can ferment various sugars [28], glucose and fructose are preferred, while sucrose is poorly metabolised without prior hydrolysis.

In addition, the optical density (OD_600_) of the culture increased from 2.26 ± 0.1 (raw molasses) to 2.74 ± 0.1 (pretreated), indicating improved cell growth. The yield and productivity were also improved, with the yield increasing from 0.76 to 0.81 g/g and the productivity increasing from 0.26 to 0.45 g/L·h. Previous studies reported that molasses hydrolysis significantly improves both the efficiency and kinetics of lactic acid fermentation [10,17,40]. While *L. fermentum* ATCC 9338 demonstrated strong performance in this study, other LAB strains might exhibit comparable or even superior fermentation efficiency when applied to pretreated sugarcane molasses. Strains such as *L. plantarum*, *L. rhamnosus*, and *L. casei* have been reported to efficiently convert sugar-rich hydrolysates into lactic acid [20,41]. Investigating the behaviour of these strains under similar conditions could broaden the applicability of this bioprocess and support the identification of additional candidates suitable for large-scale fermentation.

### 3.3. Optimisation of Lactic Acid Production from Pretreated Molasses Using Box–Behnken Design

An analysis of the effect of three potential independent variables (initial sugar concentration, inoculation rate, and stirring speed) for lactic acid production by *Limosilactobacillus fermentum* ATCC with pretreated molasses was conducted using different combinations in a total of 15 runs, and the corresponding results are presented in Table 4.

#### 3.3.1. Statistical Analysis of the Box–Behnken Model

The influence of the initial sugar concentration (X_1_), inoculation rate (X_2_), and stirring speed (X_3_) on lactic acid production was evaluated using a Box–Behnken design with 15 experimental runs. The regression model for lactic acid concentration was highly significant, as shown in Table 5. Among the linear terms, the stirring speed (X_3_) had the strongest effect, followed by the initial sugar concentration (X_1_) and the inoculation rate (X_2_). All the interaction terms and quadratic effects were also highly significant (*p* < 0.0001), indicating strong curvature and interactions between the variables.

The model showed excellent predictive ability with an R^2^ of 0.9999, an adjusted R^2^ of 0.9998, and a predicted R^2^ of 0.9991. The low residual standard deviation (0.0954) and coefficient of variation (0.23%) confirm the accuracy and reproducibility of the model. The lack of fit test was insignificant (*p* = 0.299), supporting the adequacy of the model.

**Figure 3:**
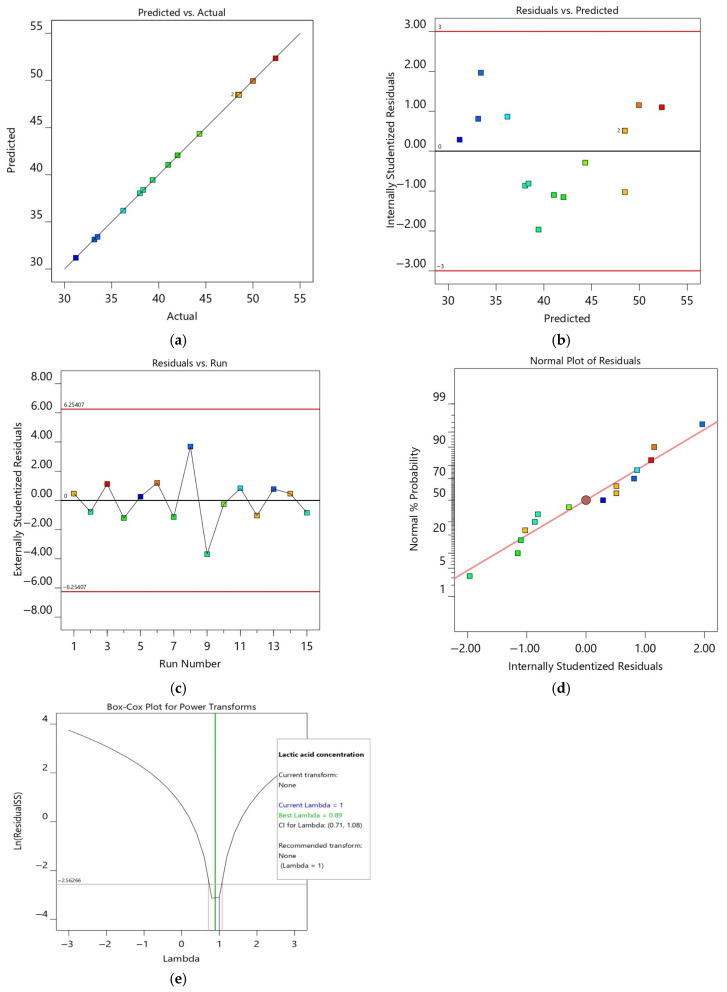
Model diagnostic plots for lactic acid production optimisation: (**a**) Predicted vs. Actual (**b**) Residual vs. Predicted (**c**) Residual vs. Run (**d**) Normal Probability Plot of Residuals (**e**) Box–Cox Plot. Colors correspond to individual experimental runs and are used consistently across plots for visual tracking.

The model diagnostics (Figure 3) further confirm a normal distribution of residuals, minimal variance, and no significant outliers. The Box–Cox plot indicated that no transformation was required (λ = 1), supporting the use of the original dataset.

#### 3.3.2. Interactive Effects of the Fermentation

**Figure 4:**
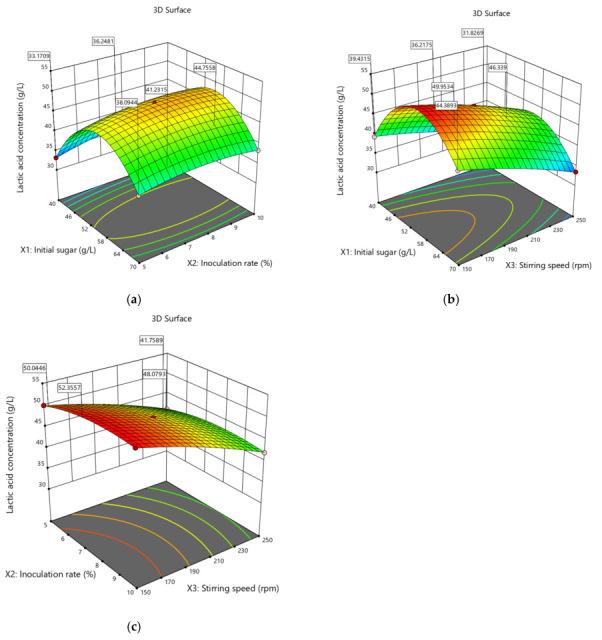
3D response surface plots showing interactive effects of process variables on lactic acid production: (**a**) Initial sugar concentration and inoculation rate; (**b**) Initial sugar concentration and stirring speed; (**c**) Inoculation rate and stirring speed. The surface color gradient represents lactic acid concentration (g/L), with red/yellow indicating higher and green/blue lower concentrations.

The 3D response surface plots (Figure 4) illustrate the interaction effects of the three variables on the lactic acid concentration. In the case of the initial sugar concentration and the inoculation rate (Figure 4a), the lactic acid concentration increased with the sugar concentration up to 55 g/L, after which it plateaued or decreased, possibly due to osmotic stress or substrate inhibition. Previous studies [42,43] reported that the accumulation of organic acids like lactic, acetic, ethanol, or succinic acid can inhibit microbial activity, which may be mitigated by pH control in the case of a batch process [11]. Similarly, decreasing the inoculation rate to approximately 8 to 9% (compared to the initial condition, 10%) improved lactic acid production. At this value of the inoculation rate, competition for resources such as nutrients and oxygen between cells is minimised, allowing for more efficient substrate conversion and enhanced cell density [44].

The interaction between the initial sugar concentration and the stirring rate (Figure 4b) showed that higher sugar concentrations and moderate stirring speeds (under 200 rpm) favoured lactic acid production. At excessively high stirring speeds, reduced performance was observed, probably due to the shear stress acting on the bacterial cells [45].

The inoculation rate and stirring speed interaction (Figure 4c) showed that increasing both variables improved the lactic acid concentration up to an optimum point. Beyond this, higher stirring rates showed diminishing returns, as moderate agitation enhances mass transfer while avoiding shear stress that can damage cell membranes and reduce viability, as reported by [46].

#### 3.3.3. Validation of the Model

To validate the model, three independent experiments were conducted under the optimal conditions predicted by the Box–Behnken design: a 55.67 g/L initial sugar concentration, an 8.85% inoculation rate, and a stirring speed between 150 and 153.33 rpm (Table 6). The observed lactic acid concentrations (51.5–52.4 g/L) were in excellent agreement with the predicted values (52.6–52.8 g/L), with residuals ranging from −1.238 to −0.239 g/L and relative errors below 2.5%. Among the three runs, the second assay yielded the highest lactic acid concentration (52.359 g/L) and the lowest prediction error (0.454%), confirming the accuracy of the model. Although the software suggested a stirring speed of 153.33 rpm, this value may be impractical to implement with standard equipment. Therefore, a rounded and more convenient stirring speed of 150 rpm is recommended for future applications.

Thus, the validated optimal conditions for lactic acid production are a 55.67 g/L initial sugar concentration, an 8.85% inoculation rate, and a 150 rpm stirring speed. These results confirm the validity and robustness of the optimisation model.

#### 3.3.4. Fermentation Kinetics Under Optimal Conditions

**Figure 5:**
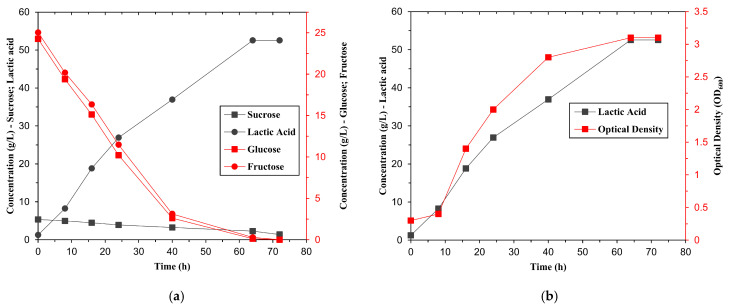
Fermentation kinetics under optimised conditions: (**a**) Time-course of sugar consumption and lactic acid production; (**b**) Effect of lactic acid accumulation on optical density of the culture.

To evaluate the kinetic behaviour of the fermentation process under the optimised conditions (55.7 g/L initial sugar concentration, 8.85% inoculation rate, and 150 rpm stirring speed), a batch experiment was carried out over 72 h. Figure 5a shows the time profile of the total sugar consumption and lactic acid production, while Figure 5b shows the effect of lactic acid accumulation on microbial growth, monitored using the optical density (OD_600_).

The results show that the sugar consumption declined rapidly in the first 24–36 h, corresponding to the exponential growth phase of the *Limosilactobacillus fermentum* strain. The lactic acid production increased proportionally, reaching a plateau after 56 h, indicating the transition to the stationary phase [47]. The final lactic acid concentration exceeded 52 g/L, closely matching the predicted value from the model.

The optical density increased steadily during the early phase but decreased slightly as lactic acid accumulated, confirming that product inhibition begins to affect biomass above 40 g/L lactic acid. These results are consistent with known lactic acid toxicity thresholds in homo- and heterofermentative Lactobacillus strains and suggest that in situ product removal could further improve productivity at scale [10,22,48].

#### 3.3.5. Determination of Specific Rotational Power

The measured optical rotation (average of six measurements) was −0.0348°, corresponding to a specific optical rotation of [α]D = −0.0139°, which translates to an enantiomeric excess of the D enantiomer of ee = −0.5351%, indicating a nearly racemic mixture with a very slight excess of the D-(lactic) enantiomer [37,38]. These results are consistent with the known metabolic behaviour of *Limosilactobacillus fermentum* ATCC 9338, a heterofermentative lactic acid bacterium capable of producing both D- and L-isomers from mixed sugar substrates [48].

While racemic lactic acid is typically produced via chemical synthesis, this route is economically unviable [49]. In contrast, our results demonstrate that low-optical-purity lactic acid can be effectively produced through fermentation using low-cost molasses, offering a greener and more viable alternative for non-polymer applications. The near-racemic nature of the product makes it particularly suitable for the synthesis of amorphous poly (lactic acid), which exhibits high biodegradability at end-of-life and enhanced flexibility compared to semi-crystalline PLA derived from optically pure monomers [50]. Furthermore, racemic LA has valuable uses in pharmaceutical, cosmetic, and biomedical formulations due to its improved solubility, favourable degradation kinetics, and pH modulation properties, especially in drug delivery systems and topical applications [49,51,52].

Using sugarcane molasses alongside the strain’s non-stereoselective microbial activity enables the sustainable and scalable production of racemic lactic acid. These results highlight the potential of agricultural by-products to support circular bioeconomy strategies in environments with limited resources, such as Côte d’Ivoire.

### 3.4. Cost Analysis of Molasses as Carbon Source

Based on the comparative sugar cost estimation method described in Section 2.7, a quantitative evaluation was performed to assess the relative economic feasibility of molasses against conventional and alternative substrates used in lactic acid fermentation. Molasses, a by-product of the sugar refining process, represents a locally abundant and low-cost carbon source for lactic acid fermentation in Côte d’Ivoire. Traditionally undervalued and used as animal feed or exported at minimal profit, molasses offers significant untapped potential for biotechnological valorisation [8,21]. Its high fermentable sugar content (45 to 55% after hydrolysis) and compatibility with simple pretreatment protocols make it particularly suitable for microbial fermentation in resource-constrained settings [53].

Based on average regional market data, raw molasses in Côte d’Ivoire is priced at USD 30–50 per tonne, equivalent to an effective sugar cost of less than USD 0.10/kg. This is five to six times cheaper than refined glucose (USD 1.25/kg) and three to four times less than imported corn syrup (USD 0.65–0.70/kg), underscoring its economic advantage (Table 7). When combined with low-energy acid hydrolysis and the nutrient-rich content of molasses, often eliminating the need for costly nitrogen supplementation, it offers a highly competitive fermentation substrate [54].

In comparison, other studies have evaluated agro-industrial residues such as cassava peels, sorghum waste, pineapple peel, cashew apple juice, and cocoa pod husks as carbon sources for fermentation [21,22,55,56,57]. While promising, many of these feedstocks require more intensive pretreatment or nutrient supplementation, reducing cost-effectiveness at scale. Molasses, in contrast, integrates easily into batch fermentation systems, aligns with circular economy principles, and supports small- to medium-scale lactic acid production without reliance on imported sugars or synthetic media [9]. These findings confirm that molasses-based fermentation can be technically viable and economically favourable for developing decentralised lactic acid biorefineries in West Africa, contributing to import substitution, waste reduction, and local bioeconomy development.

**Table 7:** Comparative cost estimate of carbon sources after pretreatment for lactic acid production.

Substrate	Sugar Content (% *w*/*w*)	Local Price (USD/ton)	Effective Sugar Cost (USD/kg)
Raw molasses	45–55	30–50	0.08–0.10
Refined glucose	>98 [58]	1250	1.25
Corn syrup	70–80 [59]	400–600	0.65–0.70
Cassava peel hydrolysate	30–40 [55]	80–100	0.25–0.30
Cocoa pod husks	20–30 [22,60]	60–80	0.25–0.35
Cashew apple juice	30–35 [21,61]	80–90	0.25–0.30
Sorghum waste	16–22 [57]	60–80	0.35–0.45
Pineapple peel	15–25 [56]	50–80	0.25–0.45

## 4. Conclusions

This research demonstrates the technical and economic feasibility of producing racemic lactic acid from Ivorian sugarcane molasses using *Limosilactobacillus fermentum* ATCC 9338 under optimised batch fermentation conditions. Acid hydrolysis pretreatment significantly enhanced the fermentable sugar content of molasses, while optimisation using response surface methodology identified the critical influence of the stirring speed and sugar concentration, leading to maximum lactic acid production.

The findings support the potential of sugarcane molasses as a sustainable and low-cost feedstock for lactic acid fermentation, aligning well with circular bioeconomy objectives by valorising agro-industrial residues. The racemic nature of the lactic acid produced extends its applicability to a wide range of non-stereospecific uses, including pharmaceuticals, cosmetics, and biodegradable materials.

Future work should focus on detailed techno-economic evaluations and the development of cost-effective and eco-friendly downstream recovery methods adapted to local contexts, particularly in resource-limited regions such as Côte d’Ivoire. Such approaches can contribute to reducing import dependency, promoting small-scale bioproduction, and supporting inclusive bioeconomic growth in developing countries like Côte d’Ivoire.

## Figures and Tables

**Table 1:** Factors and levels used in the Box–Behnken design matrix for the lactic acid production from molasses by *Limosilactobacillus fermentum* ATCC 9338.

Factors	Symbol	Levels		
		Low (−1)	Intermediate (0)	High (+1)
Initial sugar (g/L)	X_1_	40	55	70
Inoculation rate (%)	X_2_	5	7.5	10
Stirring speed (rpm)	X_3_	150	200	250

**Table 2:** Characteristics of Ivorian sugarcane molasses, before and after pretreatment (only this work).

Component	Raw Molasses	Pretreated Molasses
Brix (°Bx)	84.0 ± 1.5	76.5 ± 1.8
Dry Matter (%)	79.0 ± 2.1	72.3 ± 2.5
Sucrose (g/L)	56.80 ± 2.5	7.40 ± 0.7
Glucose (g/L)	8.60 ± 0.45	31.50 ± 1.5
Fructose (g/L)	10.90 ± 0.62	33.80 ± 1.2
Total fermentable sugars (g/L)	76.30 ± 3.1	72.60 ± 2.9
Ash (%)	12.7 ± 0.8	9.2 ± 0.6
pH	5.7 ± 0.2	5.1 ± 0.1
Crude proteins (%)	5.65 ± 0.29	5.38 ± 0.3
Calcium (mg/L)	0.60 ± 0.05	0.17 ± 0.02
Magnesium (mg/L)	0.43 ± 0.03	0.10 ± 0.02
Sodium (mg/L)	0.08 ± 0.01	0.03 ± 0.01
Potassium (mg/L)	1.82 ± 0.12	0.58 ± 0.07
Iron (mg/L)	0.70 ± 0.05	0.21 ± 0.04
Copper (mg/L)	17.0 ± 1.3	1.19 ± 0.12
Manganese (mg/L)	52.0 ± 3.5	8.0 ± 0.6
Zinc (mg/L)	19.0 ± 2.1	2.2 ± 0.3

**Table 3:** Comparison of parameters of lactic acid production by *Limosilactobacillus fermentum* ATCC 9338 using raw and pretreated molasses for 72 h.

Carbon Source	Sugar Consumption (g/L)	Optical Density (OD_600_)	Lactic Acid Production (g/L)	Productivity (g/L·h)	Yield (g/g)
Raw molasses	23.3 ± 0.31	2.26 ± 0.1	18.54 ± 0.05	0.26	0.76
Pretreated molasses	40.0 ± 0.26	2.74 ± 0.1	32.42 ± 0.03	0.45	0.81

**Table 4:** Experimental design of process variables and values of experimental data for lactic acid production by *Lactobacillus fermentum* ATCC 9338.

Run	Processes Parameters			Response
	Initial Sugar (g/L)	Inoculation Rate (%)	Stirring Speed (rpm)	Lactic Acid (g/L)
1	55	7.5	200	48.52
2	40	5	200	33.17
3	55	7.5	200	48.40
4	70	7.5	150	44.32
5	55	10	250	42.00
6	70	10	200	38.35
7	55	7.5	200	48.40
8	55	10	150	52.40
9	70	7.5	250	33.50
10	40	10	200	36.23
11	40	7.5	150	39.35
12	70	5	200	38.00
13	55	5	250	41.00
14	55	5	150	50.00
15	40	7.5	250	31.20

**Table 5:** Regression model and ANOVA for lactic acid production by *Limosilactobacillus fermentum* ATCC 9338.

Source		Sum of Squares	df	Mean Square	F-Value	*p*-Value	Significance
Model		637.65	9	70.85	7781.43	<0.0001	significant
X_1_-Initial sugar concentration		25.28	1	25.28	2776.07	<0.0001	
X_2_-Inoculation rate		5.80	1	5.80	636.68	<0.0001	
X_3_-Stirring speed		184.03	1	184.03	20,212.20	<0.0001	
X_1_X_2_		1.84	1	1.84	201.65	<0.0001	
X_1_X_3_		1.78	1	1.78	195.74	<0.0001	
X_2_X_2_		0.4900	1	0.4900	53.82	0.0007	
X_1_^2^		417.22	1	417.22	43,823.10	<0.0001	
X_2_^2^		6.96	1	6.96	763.91	<0.0001	
X_3_^2^		1.90	1	1.90	208.77	<0.0001	
Residual		0.0455	5	0.0091			
Lack of fit		0.0359	3	0.0120	2.49	0.2990	not significant
Pure error		0.0096	2	0.0048			
Cor total		637.69	14				
	Std. Dev. = 0.0954			R^2^ = 0.9999			
	Mean = 41.66			Adjusted R^2^ = 0.9998			
	C.V. % = 0.2291			Predicted R^2^ = 0.9991			
	PRESS = 0.5964			Adeq Precision = 271.6108			

**Table 6:** Results of the validation test.

Assays	Initial Sugar Concentration (g/L)	Inoculation Rate (%)	Stirring Speed (rpm)	Desirability	Observed Value (g/L)	Predicted Value (g/L)	Residual	Error (%)
1	55.72	8.85	150.14	1.000	51.595 ± 0.15	52.833	−1.238	2.343
2	55.67	8.85	153.33	1.000	52.359 ± 0.08	52.598	−0.239	0.454
3	55.90	8.38	153.57	1.000	51.457 ± 0.18	52.611	−1.154	2.193

## Data Availability

The original contributions presented in the study are included in this article; any request for further information can be addressed to the corresponding author.
